# Cellular senescence contributes to radiation-induced hyposalivation by affecting the stem/progenitor cell niche

**DOI:** 10.1038/s41419-020-03074-9

**Published:** 2020-10-14

**Authors:** Xiaohong Peng, Yi Wu, Uilke Brouwer, Thijmen van Vliet, Boshi Wang, Marco Demaria, Lara Barazzuol, Rob P. Coppes

**Affiliations:** 1 Department of Biomedical Sciences of Cells & Systems, University Medical Center Groningen, University of Groningen, Groningen, The Netherlands; 2 Department of Radiation Oncology, University Medical Center Groningen, University of Groningen, Groningen, The Netherlands; 3 Laboratory of Cellular Senescence and Age-related Pathologies, European Research Institute for the Biology of Aging (ERIBA); University Medical Center Groningen, University of Groningen, Groningen, The Netherlands

**Keywords:** Radiotherapy, Adult stem cells

## Abstract

Radiotherapy for head and neck cancer is associated with impairment of salivary gland function and consequent xerostomia, which has a devastating effect on the quality of life of the patients. The mechanism of radiation-induced salivary gland damage is not completely understood. Cellular senescence is a permanent state of cell cycle arrest accompanied by a secretory phenotype which contributes to inflammation and tissue deterioration. Genotoxic stresses, including radiation-induced DNA damage, are known to induce a senescence response. Here, we show that radiation induces cellular senescence preferentially in the salivary gland stem/progenitor cell niche of mouse models and patients. Similarly, salivary gland-derived organoids show increased expression of senescence markers and pro-inflammatory senescence-associated secretory phenotype (SASP) factors after radiation exposure. Clearance of senescent cells by selective removal of p16Ink4a-positive cells by the drug ganciclovir or the senolytic drug ABT263 lead to increased stem cell self-renewal capacity as measured by organoid formation efficiency. Additionally, pharmacological treatment with ABT263 in mice irradiated to the salivary glands mitigates tissue degeneration, thus preserving salivation. Our data suggest that senescence in the salivary gland stem/progenitor cell niche contributes to radiation-induced hyposalivation. Pharmacological targeting of senescent cells may represent a therapeutic strategy to prevent radiotherapy-induced xerostomia.

## Introduction

Xerostomia is a severe side effect of radiotherapy for head and neck cancer patients due to co-irradiation of the salivary glands^1,2^. Multiple cellular mechanisms are involved in radiation-induced salivary gland damage; however, the fundamental cellular and molecular mechanisms underlying the lasting loss of regenerative potential of salivary glands after irradiation remain to be fully elucidated.

Cellular senescence is a permanent state of cell cycle arrest^3^ induced by many pro-aging stressors, including radiation-induced DNA damage. Irradiation is known to induce senescence in both normal tissue and cancer cells after exposure to high^4^ or low doses^5^. Through secretion of a range of cytokines, chemokines, growth factors, and other signaling molecules known as the senescence-associated secretory phenotype (SASP)^6^, senescent cells can have a detrimental effect on the surrounding healthy cells. As such senescent cells have been recently shown to contribute to the development of many age-related diseases, including pulmonary fibrosis^7^, neurodegeneration^8^, atherosclerosis^9^, osteoarthritis^10^, and malignant and benign diseases^11^. Radiation has been suggested to affect surrounding non-irradiated cells through the communication with irradiated cells by SASP factors^12^. This radiation-induced bystander phenomenon is known to affect the surrounding microenvironment through gap junctions and secreted factors^13,14^, and mediate a variety of cellular effects, such as cellular senescence, cell proliferation, and malignant transformation. Accordingly, the clearance of senescent cells using genetic^15^ or pharmacological approaches^9^ can rejuvenate hematopoietic stem cells and increase the life span of aging mice^16^. However, whether the radiation altered microenvironment affects the stem cell pool and its subsequent regenerative potential remains to be elucidated.

Although radiation leads to a severe loss of acinar cells, the ductal compartment, where the salivary gland stem/progenitor cells have been suggested to reside^17^ remains after irradiation^18^ albeit with evidence of persistent DNA damage and some ductal cells undergoing cellular senescence^19^. Recent findings suggest that, in mice, reduction of quality but not quantity of stem cells is associated with aging^16^. Indeed, stem cell senescence, for instance of hematopoietic stem cells and mesenchymal stem cells^16,20^, can impair tissue homeostasis.

Salivary gland stem/progenitor cells (SGSCs) are multipotent cells that reside in the ductal compartment and can proliferate and differentiate into acinar cells which can produce saliva^21^. Senescence of SGSCs may play a role in the permanent radiation-induced salivary gland hypofunction. Therefore, it is important to understand how senescent cells affect the stem cell niche and hence the subsequent lack of regenerative potential. Moreover, whether senescent cells can act as a therapeutic target to improve salivary gland function still has to be established.

This study investigates the role of cellular senescence in radiation-induced salivary gland damage. Here, we showed accumulation of senescent cells after irradiation in or near the SGSC niche in both salivary glands and SGSC-derived organoids coinciding with upregulated SASP gene expression. Selective genetic or drug-mediated elimination of senescent cells improved the self-renewal potential of SGSCs in vitro and mitigated radiation-induced hyposalivation in vivo.

## Materials and methods

### Mice

Eight- to 12-week-old female C57BL/6 mice (Envigo, Harlan, The Netherlands) and female p16-3MR mice (kindly provided by Marco Demaria) were bred in the central animal facility of the University Medical Center Groningen. The mice were maintained under conventional conditions. Animal experimental procedures were approved by the Central Committee Animal Experimentation of the Dutch government and the Institute Animal Welfare Body at the University Medical Center Groningen.

### Immunohistochemistry staining

Salivary glands were fixed with 4% formaldehyde and embedded into paraffin. Five micrometer paraffin sections were dewaxed and boiled for 8 min with pre-heated antigen retrieval buffer. Subsequently, sections were incubated with the following primary antibodies: mouse anti-*γ*H2AX (Merck, 06-636, Darmstadt, Germany), mouse anti-BCL-2 (Dako, M0887, Glostrup, Denmark), mouse anti-p16 (CINtec® Histology Kit, 9517, Mannheim, Germany), or rabbit anti-Aquaporin5 antibody (Alomone labs, AQP-005, Jerusalem, Israel). Visualization for bright field microscopy was accomplished by adding specific secondary biotin carrying antibodies, biothynlated rabbit anti-mouse (Dako, E0413), or biothynlated Swine anti-rabbit (Dako, E0431) at 1:300 dilution. Nuclear counterstaining was performed with hematoxylin. The percentage of area positive for AQP5 staining in salivary gland tissue was quantified using ImageJ on five representative fields at ×10 magnification on three mice per group.

### Isolation of mouse salivary gland cells and organoid culture

Murine submandibular salivary glands were dissected from 8- to 12-week-old female p16-3MR mice. Salivary gland cells were isolated and cultured to form organoids as described previously^22^. In short, salivary glands were mechanically and enzymatically dissociated and cultured in DMEM-12 (Gibco/Invitrogen, 11320-074, Bleiswijk, The Netherlands) medium consisting of 1% penicillin/streptomycin (Gibco, NY, USA), glutamax (2 mM; ThermoFischer Scientific, 35050038, Paisley, UK), EGF (20 ng/ml; Sigma-Aldrich, E9644, Zwijndrecht, The Netherlands), FGF2 (20 ng/ml; peprotech, 100-18B, NJ, USA), N2 (1×; Gibco, 17502-048), insulin (10 μg/ml; Sigma-Aldrich, I6634), and dexamethasone (1 μM; Sigma-Aldrich, d4902), here called minimal medium. After 3 days, primary spheres were dissociated into single cells, seeded in Matrigel and cultured in minimal medium supplemented with Y-27632 (10 μM; Abcam, ab120129, Cambridge, UK), 10% R-spondin1 conditioned medium (provided by C. Kuo), and 50% Wnt3a conditioned medium to form organoids. After 7 days organoids were passaged by dissociation into single cells and cultured as described above.

### SA-β-galactosidase staining

Organoids were collected 7 days after (sham) irradiation, fixed and stained overnight with X-Gal solution according to the manufacturer’s instructions (Merck Millipore, KAA002RF, MA, USA). Senescent cells were identified as blue-stained cells under light microscopy. The percentage of cells positive for SA-β-gal staining in salivary gland tissue was quantified using ImageJ on three representative fields at ×20 magnification on three mice per group.

### Renilla luciferase assay

The p16-3MR gene-reporter system used in this study was as previously described^23^. Briefly, p16-3MR mice carry a three molecular reporter protein (Luciferase-mRFP-HSVtk fusion protein), which is regulated by the p16 promoter (Fig. 2d). The luciferase protein can be measured using the Renilla luciferase assay. Therefore, p16-3MR mice can be used to track radiation-induced senescence in 3D cultured organoids in vitro. Organoids derived from the salivary glands of six p16-3MR mice were collected and dissociated into single cells. The Renilla luciferase assay system was used according to the manufacturer′s protocol. In total, 100,000 cells were lysed with 100 μl of 1× Renilla luciferase assay lysis buffer. For each reaction, 20 μl of cell lysate was added to a well of a 96-well plate (Greiner Bio-one, 655075, Frickenhausen, Germany). Each sample was analyzed in triplicate.

### Quantitative real-time PCR

Cells were collected at designated time points. Total cellular RNA was extracted following the manufacturer’s instructions (Qiagen, RNeasy Mini Kit, Ref 74104, Hilden, Germany) to measure expression of cell cycle genes Cdkn2a (p16^Ink4a^), Cdkn1a (p21), and SASP genes (including Il6, Mcp1 Cxcl1), and the senescence transcriptome core signature Gdnf in mouse salivary gland organoid-derived cells (3–6 mice per group) and salivary gland tissue (6 mice per group). The primer sequences are listed in Supplementary Table 1. RNA reverse transcription was performed as described previously^22^. First-strand cDNA synthesis was performed by using 500 ng total RNA, 1 μl dNTP Mix (10 mM), 1 μl random primers (100 ng), 4 μl 5× First-stand Buffer, 2 μl DTT (0.1 M), 1 μl RNase OUTTM (40 units/μl), and 1 μl M-MLV RT (200 units), 20 μl in total for each reaction volume. To measure gene expression, the SYBR assay kit (Bio-Rad) was used. Briefly, 2.5 μl cDNA was mixed with 6.25 μl SYBR Green PCR Master Mix and 3.75 μl primers mix (20 μl forward primer, 20 μl reverse primer and 1160 μl dH_2_O, primer concentration of 100 nmol) for the genes of interest. qPCR conditions were as follows: 95 °C for 3 min, 39× (95 °C for 10 s, 55 °C for 10 s, and 72 °C for 30 s), 95 °C for 10 s, 65 °C for 5 s, 95 °C for 50 s. All reactions were run in triplicate on a BIO-RAD Real-Time PCR System. All the reagents mentioned above are from Invitrogen.

### Flow cytometry

Salivary gland organoids derived from mice were harvested at designated time points and dissociated into single cells. For cell cycle analysis, after two washes with phosphate-buffered saline (PBS), cells were fixed with 70% ethanol, incubated overnight at 4 °C. Cells were collected by spinning them down for 5 min at 1000 r.p.m. at 4 °C, followed by washing with PBS. After two washes with PBS, cells were treated with 20 μl DNase free RNase A (stock concentration of 20 mg/ml) to remove residual RNA (Sigma-Aldrich) and incubated for 30 min at 37 °C. Four hundred microliters of propidium iodide solution (50 μg/ml) was added to cells and incubated for 1 h at room temperature. For CD24/CD29 population analysis, Pacific Blue^™^ anti-mouse CD24 (BioLegend, 101820, CA, USA) and FITC anti-rat CD29 (BD Biosciences, 555005, NJ, USA) antibody incubations were performed at room temperature for 15 min, followed by a wash step in PBS. Finally, propidium iodide solution (1 μg/ml) was added to the cells. Samples were analyzed using the XDP flow cytometry machine. Data were analyzed by FlowJo software.

### SASP experiments with conditioned medium

Organoids cultured in WRY medium were (sham-) irradiated at day 5 (D5) in culture. Medium was collected at D12 and mixed with fresh medium in a 1:1 ratio, resulting in control (C50%) and IR (IR50%)-conditioned medium. 1 × 10^4^ fresh single SGSCs released from passage 2 organoids were cultured with conditioned medium. Seven days later, Matrigel was dissolved using Dispase and organoid formation efficiency (OFE) was calculated as mentioned previously^22^.

### In vitro irradiation and treatment with ganciclovir (GCV) and ABT263

The irradiation assay was performed as described previously^24^. In short, photon irradiation was performed using a Cesium-137 source with a dose rate of 0.59 Gy/min. All irradiation experiments were performed on 5-day-old organoids cultured in 12-well plates derived from at least three mice per group. Five-day-old organoids were irradiated with 7 Gy. Seven days later, 10 μg/ml GCV (Sigma-Aldrich, G2536) or 0.313 μM ABT263 (Selleckchem, Cat No. S1001, TX, USA,) were administrated to irradiated organoids and sham-irradiated control cells were incubated, while the same volume of vehicle solvent of GCV or ABT263 were added to the controls. Organoids were collected and dissociated into single cells, and reseeded to next passage at 1 × 10^4^ cell density as mentioned previously. OFE was calculated 7 days later.

### In vivo irradiation and treatment with ABT263

The irradiation experimental setup employed here was as described previously^24^. In short, the salivary glands of 2–3-month-old female C57BL/6 mice were (sham-) irradiated with a single dose of 15 Gy X-rays (Precision X-ray). Eight weeks after irradiation, mice were treated with vehicle (ethanol:polyethylene glycol 400:Phosal 50 PG at 10:30:60) or ABT263 (in ethanol:polyethylene glycol 400:Phosal 50 PG at 10:30:60) by oral gavage at 50 mg/kg per day for seven consecutive days for two cycles with a 2 week interval in between. Saliva production was measured as previously described^25^. Briefly, after stimulation with pilocarpine (2 mg/kg), saliva was collected for 15 min at baseline, 7, 13, and 22 weeks after irradiation and/or ABT263 treatment. This in vivo experiment was performed using eight mice per group.

### Statistical analysis

GraphPad Software version 8 was used for all statistical analyses. Two-tailed Student’s *t*-test, Wilcoxon signed-rank test, and two-way ANOVA were used to estimate statistically significant differences between groups. Investigators were blinded to allocation during in vivo experiments and outcome assessments. All values were represented as means ± s.e.m. Numbers (*n*) for tested groups are stated in the figure legends. All *p* values were two-sided. *P* < 0.05 was considered to be statistically significant. All replicates in this study were samples from different mice.

## Results

### Radiation induces senescence in mouse and human salivary glands

**Fig. 1:**
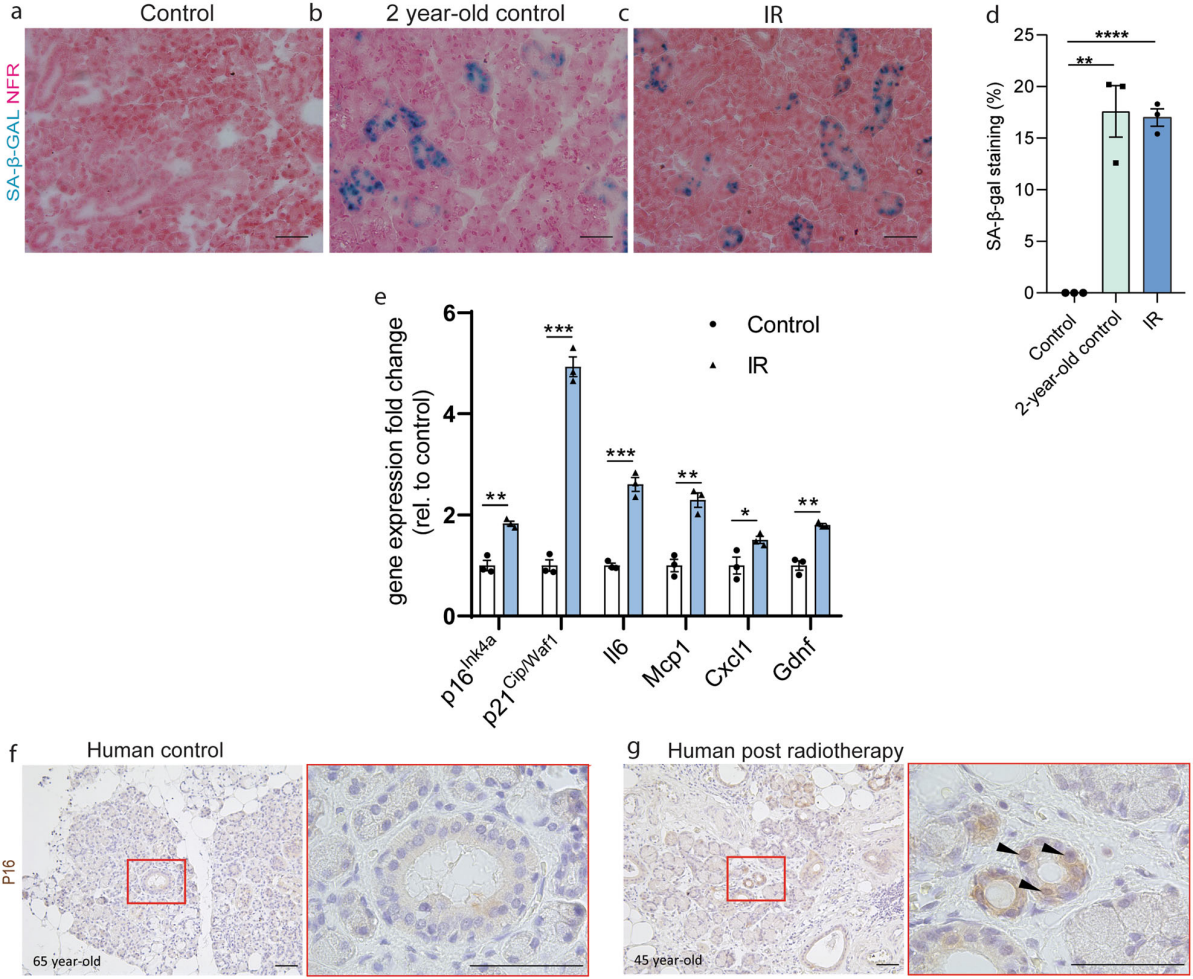
Cellular senescence in irradiated mouse and human salivary glands. **a**–**d** Representative images of SA-β-gal (blue) staining in mouse salivary gland tissue from **a** control (14-week-old), **b** 2-year-old control, **c** 8 weeks post 15 Gy irradiation (14-week-old), and **d** quantification of SA-β-gal-positive cell percentage, *N* = 3 mice/group. Student’s *t*-test. **e** RT-qPCR analysis of the expression of senescence markers in salivary gland tissue of control and 15 Gy irradiated mice, *N* = 3 mice/group. Multiple Student’s *t*-test. **f**, **g** Representative images of p16 (brown) of human control (65-year-old) (**f**) and radiation damaged (45-year-old) (**g**) salivary glands. Scale bar, 100 µm. Data are mean ± s.e.m., *p < 0.05; **p < 0.01, ***p < 0.001, ****p < 0.0001.

To determine whether radiation induces senescence in salivary glands, submandibular glands (SGs) of control, 2-year-old, and 8 weeks post 15 Gy irradiated mice (IR) were stained for senescence-associated β-galactosidase (SA-β-gal) (Fig. 1). High levels of SA-β-gal were observed in both 2-year-old and irradiated SGs, whereas SGs of sham-irradiated control mice were negative for SA-β-gal (Figs. 1a–d). Interestingly, SA-β-gal expression was only observed in the striated and excretory ducts, which have been suggested to contain the mouse SGSCs^17,21,26^. Moreover, SG cells isolated from mice at 8 weeks post IR displayed increased expression of senescence-associated genes, including the cell cycle regulators p16^Ink4a^ (also known as Cdkn2a) and p21^Cip1/Waf1^ (Cdkn1a), the pro-inflammatory factors Il6, Mcp1, Cxcl1, and the senescence transcriptome core signature Gdnf^27^ (Fig. 1e). A similar ductal staining pattern was observed in human SG samples obtained from a 45- and 65-year-old irradiated patient (IR) but not in a 63, 65, and 85-year-old unirradiated patient (control), as indicated by the increased presence of p16-positive cells in the main ducts (Fig. 1f–g and Supplementary Fig 1a). These data indicate that in SGs senescence can be induced by both aging and radiation, being most abundantly present in the region thought to contain the putative SG somatic stem cells. Interestingly, in salivary glands, BCL-2 is expressed in the striated and excretory ducts^28^ where the salivary gland stem cells have been suggested to reside^17,26^ and may be related to resistance to apoptosis. Therefore, we verified the expression of BCL-2 in the salivary gland striated and excretory ducts as shown in Supplementary Fig. 1b.

### Senescence and SASP factors are elevated in irradiated salivary gland organoids

**Fig. 2:**
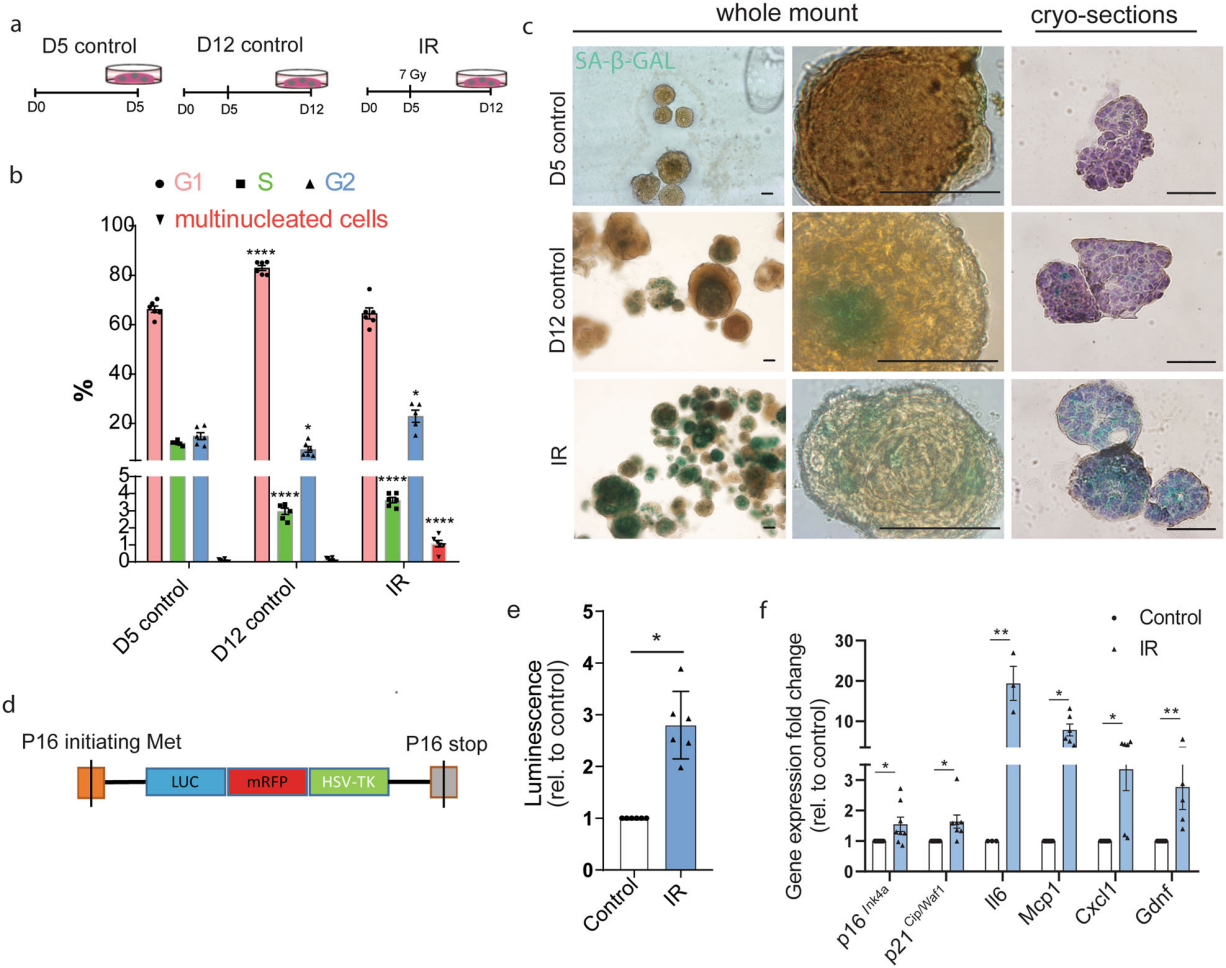
Induction of radiation-induced cellular senescence in salivary gland organoids. **a** Experimental design. **b** Cell cycle distribution of cells derived from 5-day-old (D5), D12 control organoids, and 7 Gy-irradiated (IR) organoids. Data represent the percentage of cells in G1, S, and G2 phases of the cell cycle and being multinucleated. *N* = 6/group. **c** Representative images of SA-β-gal staining performed on whole mount and cryo-sections of organoids collected at the indicated times. **d** Scheme of the p16-3MR mouse model. Experiments were performed as in panel **a. e** Luminescence measurements of cells isolated from D12 control and IR organoids. *N* = 6/group. **f** RT-qPCR analysis of the expression of senescence markers in D12 control and IR organoids. Fold change as compared to control of the levels of the indicated genes relative to Ywhaz. p16^Ink4a^: *N* = 8/group; p21^Cip/Waf1^, Mcp1, Cxcl1: *N* = 6/group; Il6: *N* = 3/group; Gdnf: *N* = 5/group. Data are mean ± s.e.m. *p < 0.05; **p < 0.01; ****p < 0.0001, Student’s *t*-test (**b**), Wilcoxon signed-rank test (**e**, **f**). Compared to D5 controls (**b**) or D12 controls (**e**, **f**). Scale bar, 50 µm.

To further study the role of radiation-induced senescence, we used our previously developed mouse SG organoid model. These organoids contain SGSCs capable of giving rise to all major SG cell types^21,29^. Five-day-old (D5) organoids were irradiated with 7 Gy and analyzed 7 days later (D12), a dose and a time known to induce senescence in vitro^30^. As controls, unirradiated D5 and D12 organoids were used (Fig. 2a).

First, cell cycle distribution of the irradiated organoid-derived cells was assessed (Fig. 2b and Supplementary Fig. 2a–c). The D12 control compared to the D5 control organoids showed an increase in the percentage of cells in G1 accompanied with a decrease in S and G2 indicating a decrease in cell proliferation with time in culture. After IR an increase in the percentage of cells in G2 was observed accompanied with a decrease of cells in S, and an increase in multinucleated cells (Supplementary Fig. 2a–c) likely due to radiation-induced G2 arrest and aberrant cell cycle progression^31^.

Next, we analyzed the expression of stem cell enriched markers CD24/CD29 (ref. ^29^) in organoid cells derived from irradiated and unirradiated organoids (Supplementary Fig. 3). Indeed, CD24^hi^/CD29^hi^ is abundantly expressed in our organoids at 5 days of culture but decreases significantly at D12 with or without irradiation. Interestingly, the most differentiated population being CD24^lo^/CD29^lo^ is significantly increased after irradiation, whereas CD24^hi^/CD29^lo^ and CD24^hi^/CD29^med^ progenitor populations^29^ are increased in both control and irradiated D12 organoids. These data show a shift from stem cells to more differentiated cells after prolonged culture and irradiation.

Evidence of DNA damage response in the salivary gland stem/progenitor cells exposed to radiation has been previously shown in salivary gland organoids^32^ as well as in irradiated salivary gland tissue^19^. Indeed, *γ*H2AX foci indicating radiation-induced double-strand breaks were high at 0.5 h and decreased at 3 and 24 h after irradiation with 7 Gy confirming activation of the DNA damage response in our organoid system (Supplementary Fig. 2d, e).

Next, to assess the presence of cellular senescence, SA-β-gal staining was performed on whole mount organoids and organoid-derived cryo-sections (Fig. 2c). Indeed, SA-β-gal activity was increased in irradiated organoids compared to D5 and D12 control organoids. D12 control organoids did exhibit some SA-β-gal-positive cells in the center indicating the presence of endogenous cellular senescence, likely due to lack of nutrition and/or oxygen after prolonged growth (Fig. 2c and Supplementary Fig. 4a). Since it was not possible to accurately quantify the level of senescence in 3D organoids, we used organoids derived from p16-3MR transgenic reporter mice (Fig. 2d) to assess senescence based on p16 gene expression levels via luciferase expression. These mice carry a 3MR (trimodality reporter) protein under the control of the promoter for p16^INK4a^ (refs. ^23,33^). The 3MR fusion protein consists of a Renilla luciferase (LUC) for luminescence detection, a monomeric red fluorescent protein for fluorescence, and a herpes simplex virus thymidine kinase (HSV-TK), which converts GCV into a toxic DNA chain terminator causing p16 expressing senescent cells to selectively undergo apoptosis. Quantification of p16-expressing cells was as such based on the luciferase activity. Indeed, senescence-associated luciferase activity significantly increased in irradiated organoids (Fig. 2e) when compared to D12 control organoids while the total cell number in irradiated organoids decreased (Supplementary Fig. 4b, c). A significant increase in gene expression of p16, p21 and the SASP factors, Il-6, Mcp1, Cxcl1, and Gdnf further confirmed the induction of radiation-induced senescence in our SG organoid model (Fig. 2f).

### SASP factors secreted by irradiated organoids compromise SGSC self-renewal potential

**Fig. 3:**
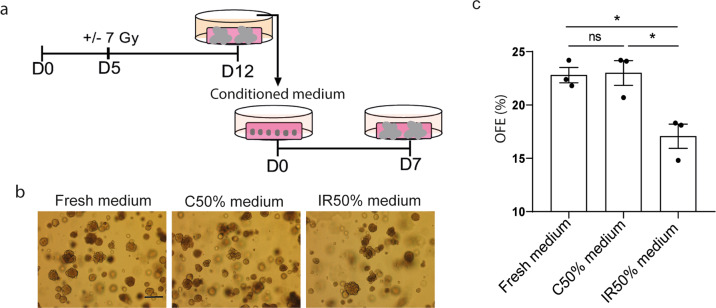
Conditioned medium of irradiated organoids reduces self-renewal potential of salivary gland stem/progenitor cells. **a** Schematic overview of the study design. **b** Representative images of passaged day 7 (D7) organoids cultured in fresh, C50% and IR50% medium and their **c** organoids formation efficiency (OFE). Medium was collected at D12 and mixed with fresh medium in a 1:1 ratio, resulting in control (C50% medium) and IR (IR50% medium) conditioned medium. Scale bar, 200 μm. *N* = 3/group, *p < 0.05; ns not significant, Wilcoxon signed-rank test.

Since the senescent cells are especially abundant in the ductal compartment (Fig. 1), which has been suggested to contain the SGSCs, we investigated the influence of SASP factors on the self-renewal potential of SGSCs. D5 SG organoids were irradiated with 7 Gy. At D12 in culture, the conditioned medium from these organoids (Fig. 3a) was collected and mixed with fresh medium in a 1:1 ratio, resulting in control mixed medium (C50% medium) and IR mixed medium (IR50% medium) (Fig. 3a). After incubation with IR50% medium, the OFE of unirradiated SG cells was significantly decreased compared to fresh medium and C50% medium (Fig. 3b, c), suggesting that the secreted factors may compromise SGSC self-renewal potential.

### Clearance of radiation-induced senescent cells by GCV or ABT263 treatment increases SG OFE

**Fig. 4:**
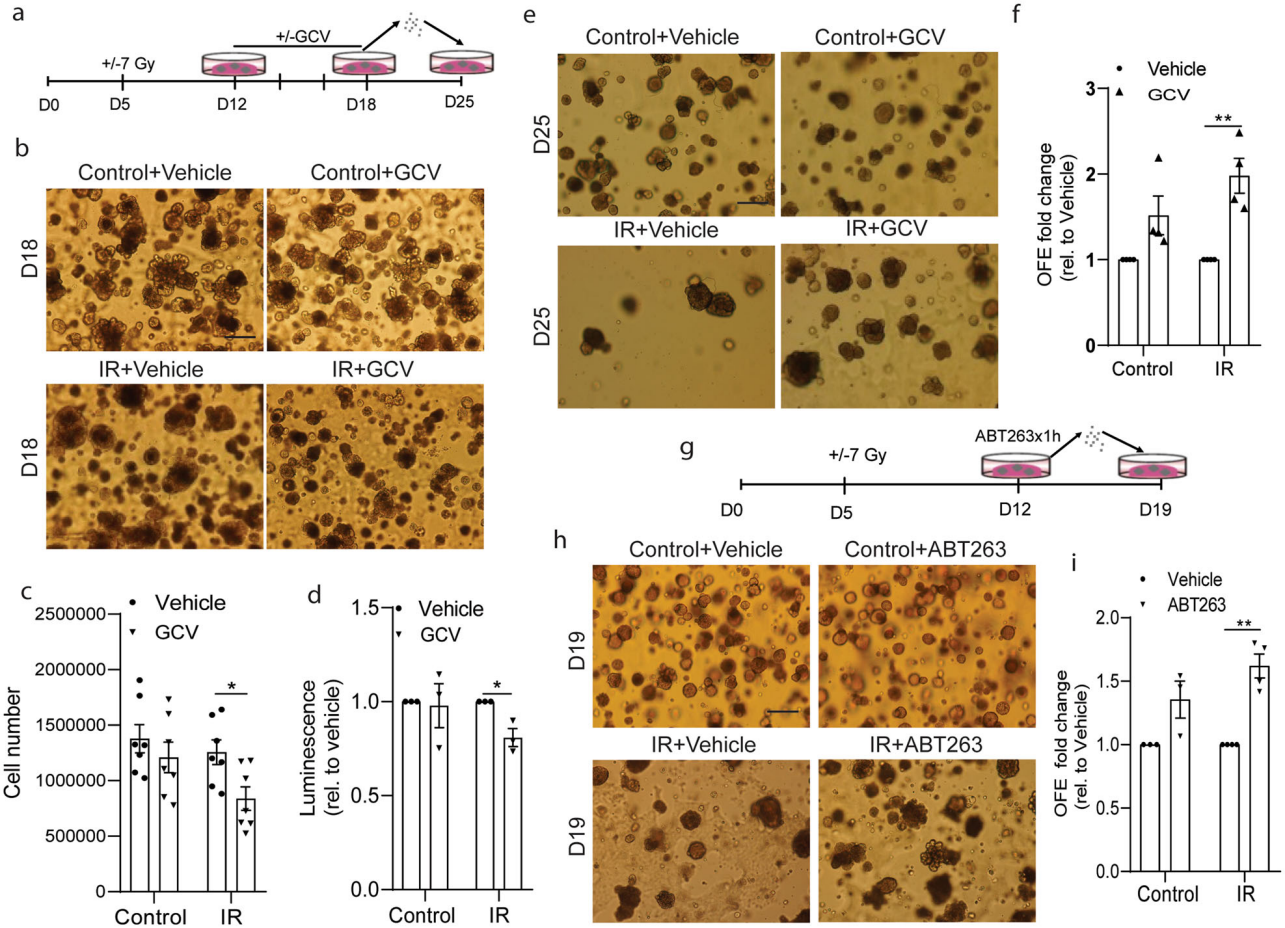
Elimination of radiation-induced senescent cells enhances SGSC self-renewal potential. **a** Experimental design for panels **b–f**. Organoids derived from p16-3MR mice were irradiated at day 5 (D5) in culture, and treated with 10 μg/ml ganciclovir (GCV) at D12. GCV was refreshed every other day for 6 days. **b** Representative images and **c** cell number of organoids at D18. *N* = 7/group. **d** Luminescence quantification based on the same number of cells derived from D18 organoids of (sham-)IR group ± GCV. *N* = 3/group. **e** Representative images of salivary gland organoids at D25. **f** Quantification of OFE at D25. *N* = 4/group. **g** Experimental design for panels **h** and **i**. Organoids derived from C57BL/6 mice (6–8 weeks old) were (sham-)irradiated at D5, and (sham-) treated with 0.313 μM ABT263 for 1 h at D12. Organoid-derived single cells were reseeded to determine OFE at D19. **h** Representative images of salivary gland organoids at D19. **i** Quantification of OFE at D19. *N* = 3/group. Data are means ± s.e.m. *p < 0.05, **p < 0.01, Student’s *t*-test (**c**), Wilcoxon signed-rank test (**d**, **f**, **i**). Scale bar, 200 μm.

Next, to further study the effect of senescent cells on SGSC function, we used GCV treatment to specifically kill senescent cells in SG organoids derived from p16-3MR mice^23^. To select the appropriate concentration, we first checked the toxicity of GCV treatment on the survival of unirradiated SGSCs (Supplementary Fig. 5a). A reduced OFE was observed at doses above 20 μg/ml GCV (Supplementary Fig. 5b, c). Therefore, we chose to treat irradiated organoids with 10 μg/ml GCV, a dose that did not affect normal organoid culture. Organoids derived from P16-3MR transgenic mice were irradiated with 7 Gy at D5 and subsequently treated with 10 μg/ml GCV at D12 in culture and refreshed every other day until D18 (Fig. 4a). GCV treatment resulted in a significant reduction in cell number (Fig. 4b, c) and p16 reporter expression (Fig. 4d) at D18. Next single cells derived from these organoids were replated to assess self-renewal potential after elimination of the senescent cells using GCV (Fig. 4a). Strikingly, cells from irradiated GCV-treated organoids showed a significant increase in OFE in the next passage compared to that of irradiated vehicle-treated organoids. A small but not significant increase was observed using cells derived from non-irradiated GCV-treated organoids (Fig. 4e, f). Next, we tested the commonly used senolytic drug ABT263 on C57BL/6 SG-derived cells. ABT263, a specific inhibitor of BCL-2 and BCL-xl, selectively kills senescent cells by inducing apoptosis^16^. Similarly to GCV, 1 h treatment with 0.313 μM ABT263 (Fig. 4g), a dose effective in killing radiation-induced senescent WI-38 fibroblasts^16^ and that does not affect normal SG organoid growth (Supplementary Fig. 6a–c), was used to remove senescent cells. SG organoid-derived cells reseeded and collected 7 days later showed a significant increase in OFE after irradiation and ABT263 treatment (Fig. 4h, i and Supplementary Fig. 6d–f). Moreover, when exposed to ABT263 for 5 or 10 h, a treatment that slightly reduced OFE in control cells (Supplementary Fig. 6a–c), OFE of irradiated cells was enhanced (Supplementary Fig. 6g–i). Cumulatively, these data indicate that clearance of senescent cells in irradiated organoids increases SGSC self-renewal potential.

### ABT263 treatment ameliorates radiation-induced hyposalivation

**Fig. 5:**
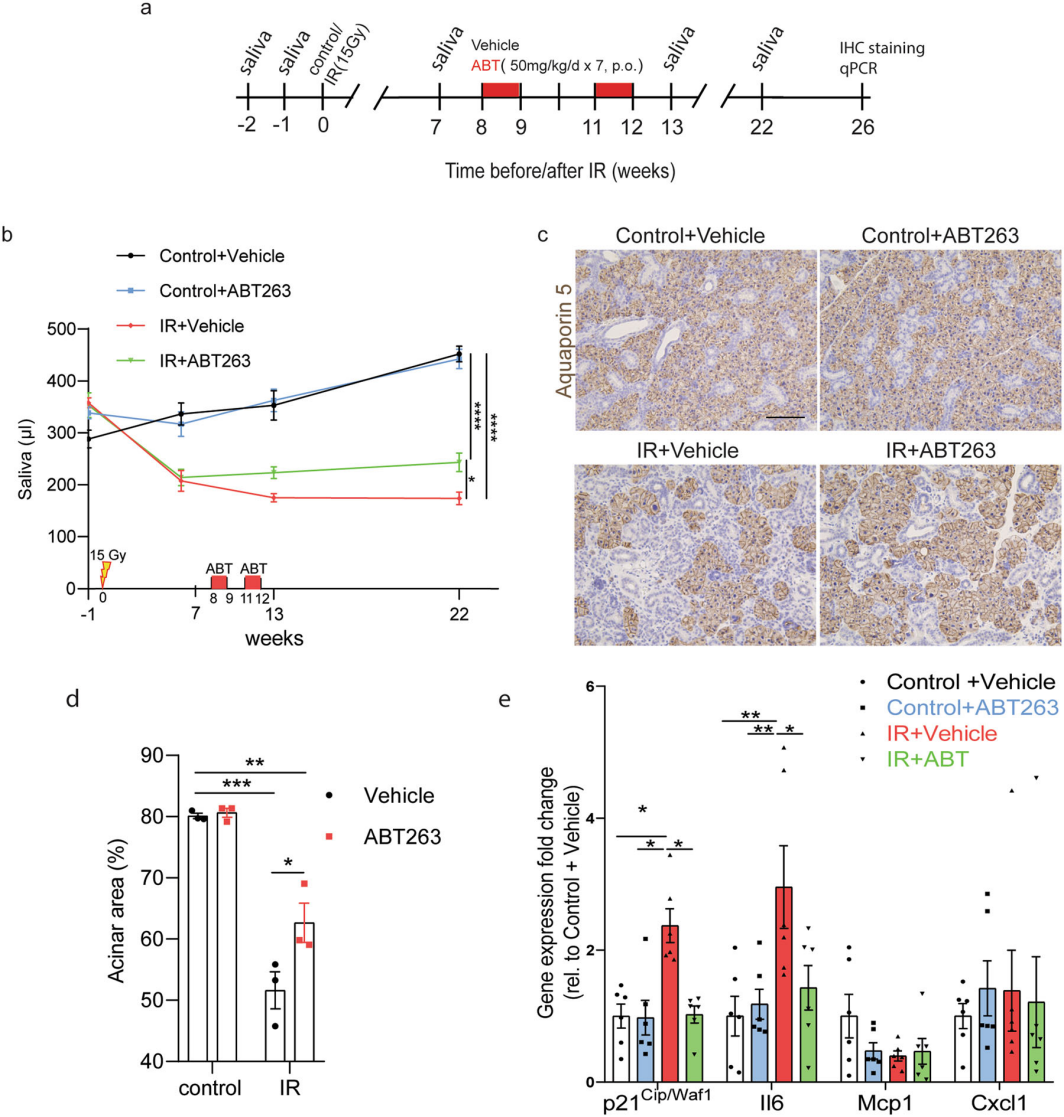
ABT263 treatment improves SG secretory function after irradiation. **a** Experimental design for panel **b**. (sham-)irradiated C57BL/6 mice were administered with vehicle or ABT263 (50 mg/kg/day × 7 days, two cycles with a 2-week interval). Saliva secretion was measured at the indicated time points for 15 min. **b** Total stimulated saliva secretion measured before irradiation (−1 week, baseline), before ABT263 treatment (7 weeks after irradiation), and after ABT263 treatment (13 and 22 weeks after irradiation). *N* = 8 mice/group. Two-way ANOVA. **c** Aqp5 staining of SGs at 26 weeks after IR with or without ABT263 treatment. Aqp5-positive cells are functional acinar cells. Scale bar, 100 µm. **d** Quantification of the percentage of acinar area relative to the total salivary gland area. Two-way ANOVA. *N* = 3 mice/group. **e** qRT-PCR analysis of cell cycle and senescence marker genes at 26 weeks after IR with or without ABT263 treatment. *N* = 6 mice/group. Student’s *t*-test. Data are means ± s.e.m., *p < 005. **p < 0.01, ***p < 0.001, ****p < 0.0001.

Next, we tested whether ABT263 treatment improved salivary gland function after irradiation in vivo. The salivary glands of C57BL/6 mice were locally irradiated with 15 Gy^34^. ABT263 was administered for 7 days by oral gavage at a dose of 50 mg/kg/day at 8 and 11 weeks post irradiation (Fig. 5a). As expected, at 13 weeks after irradiation saliva production of irradiated animals was reduced compared to control animals. Interestingly, ABT263 treatment significantly improved saliva production in irradiated animals (Fig. 5b). Moreover, we observed an increased number of Aquaporin5 (Aqp5)-positive acinar cells in the irradiated and ABT263 treatment group (Fig. 5c, d) and reduced gene expression of p21^waf1^ and Il6 (*p* < 0.01), although not of Mcp1 and Cxcl1, when compared to irradiated glands treated with vehicle (Fig. 5e). These data indicate that treatment with ABT263 can improve the morphology and function of the irradiated salivary glands.

## Discussion

Cellular senescence has recently been implicated in many age-related diseases concurrently with tissue deterioration^35,36^. Here, we showed the presence of senescent cells after aging and irradiation in murine and human salivary glands, most abundantly in the ductal compartment thought to harbor the tissue somatic stem/progenitor cells^17,21,26^. Similarly, after irradiation salivary gland-derived organoids showed a significant induction of senescence and elevated expression of SASP genes, leading to a partial decrease in SGSC self-renewal potential due to secreted SASP factors. Importantly, selective removal of senescent cells genetically or pharmacologically improved in vitro SGSC potency and enhanced in vivo salivary gland function after irradiation, indicating the close relation between radiation-induced senescence, SASP factors, and stem cell function.

The inability of SGSCs, residing in a niche containing or being close to senescent cells, to proliferate may exacerbate salivary gland damage by limiting tissue regeneration potential after injury. The observation that after aging^37^ or upon an ablative radiation dose^38^ the remaining SGSCs, when taken out of their niche and cultured as organoids, have the same regenerative potential as young/untreated SGSCs agrees with this hypothesis. Indeed, evidence of DNA damage response activation in organoids presented here and in a previously published study^32^ and in irradiated salivary gland tissue^19^ and the high level of senescence and SASP gene expression after irradiation as shown here in SGSC-derived organoids may contribute to this phenomenon.

SASP factors can disrupt the surrounding healthy cells through paracrine activity via different mechanisms, such as recruitment of inflammatory cells, remodeling of the microenvironment, induction of fibrosis, and inhibition of stem cell function, the latter recently called “senescence-stem lock model”^39^. This model showed that chronic SASP secretion can prevent tissue regeneration by locking stem cells in a state of de-differentiation^6,39,40^. Our data indicate that SASP factors inhibit the regenerative potential of SGSCs after irradiation ultimately contributing to salivary gland dysfunction. However, which SASP factors directly mediate SGSC dysfunction after irradiation remains unknown. IL1 and IL6, two main SASP factors, were recently reported to block stem cell differentiation promoting tissue aging^41,42^. Here we observed a similar increase in IL6 expression after irradiation suggesting that IL6 may play a role in radiation-induced SGSC dysfunction. Indeed, using IL6^−/−^ mice it was shown that IL6 deficiency can ameliorate but is not sufficient to rescue radiation-induced salivary hypofunction^19^. Interestingly, IL6 can attract immune cells like T cells to senescent regions^43^, and inhibit the proliferation of neural stem cells in vitro and in vivo^44^.

It has been found that the clearance of senescent cells may attenuate aging^45^ and radiation-induced premature aging^16,39^. Indeed, removal of radiation-induced senescent cells, either genetically with GCV on p16-3MR mouse derived cells or pharmacologically with ABT263, ameliorated SGSC self-renewal capacity in vitro, and improved saliva production in vivo. Moreover, ABT263 treatment successfully improved function and morphology of salivary glands after irradiation. This might be the result of a rejuvenation of the stem cells themselves and/or the restoration of the stem cell microenvironment (niche)^16,46^. Indeed, downregulation of cell cycle arrest gene p21 and most importantly the SASP gene IL6 by ABT263 treatment seems to agree with this. In salivary glands, the constitution of the stem cell niche before and after irradiation and the signals governing the stem cell niche have not been fully established. Interestingly, BCL-2 is expressed in salivary gland striated and excretory ducts where the SGSCs have been suggested to reside^17,28^, making them more resistant to radiation-induced apoptosis. Previous data showed that BCL-2 enhances hematopoietic stem cell function by anti-apoptotic action^47,48^ and mediates radio-resistance of hair follicle bulge stem cells^49^. Moreover, overexpression of BCL-2 increases quiescence of hematopoietic stem cells^50^. Taken together, these data suggest that overexpression of BCL-2 might mediate radiation-induced resistance to apoptosis but could also increase quiescence in salivary gland stem/progenitor cells. However, it is unclear to what extent BCL-2 targeted senolytics work on the quiescent stem cells while eliminating the senescent cells in vivo. Therefore, the effects of senolytics on stem cells, their niche, and the related long-term (side-)effects need further investigation.

In conclusion, this study provides evidence that senescent cells contribute to radiation-induced hyposalivation and suggests that eliminating senescent cells may represent a new therapeutic intervention for the treatment of xerostomia associated with radiotherapy. However, it should not be neglected that ABT263 has some toxic side effects in patients, such as thrombocytopenia and neutropenia^51^. Based on the current study, it is tempting to speculate that a few treatment cycles of ABT263 would be sufficient to eliminate the radiation-induced senescent cells; however, further work is needed to determine the safety, efficacy, and therapeutic window of it and other senolytic drugs.

## Supplementary information

Supplementary Table 1

Supplementary Table 1 legends

Supplementary figure legends

Supplementary figure 1

Supplementary figure 2

Supplementary figure 3

Supplementary figure 4

Supplementary figure 5

Supplementary figure 6
