# Supplementary material for: Cellular senescence contributes to radiation-induced hyposalivation by affecting the stem/progenitor cell niche
Source: Cell Death Dis. 2020 Oct 14;11(10):854. doi: 10.1038/s41419-020-03074-9 (PMC7566836; doi:10.1038/s41419-020-03074-9)
Supplement: Supplementary file 1 — Supplementary Table 1 [file 41419_2020_3074_MOESM1_ESM.docx]

| **Genes** | **Forward primer** | **Reverse primer** | **Length** |
| --- | --- | --- | --- |
| Cdkn1a(p21) | AGGCAGACCAGCCTGACAGAT (21) | TCCTGACCCACAGCAGAAGAG (21) | 111 |
| Cdkn2a(p16^Ink4a^) | GAACTCTTTCGGTCGTACCC (20) | CGAATCTGCACCGTAGTTGA (20) | 88 |
| Gdnf | CGCTGACCAGTGACTCCAATATGC (24) | TGCCGCTTGTTTATCTGGTGACC (23) | 116 |
| Il6 | ATACCACTCCCAACAGACCTGCT (23) | CAGAATTGCCATTGCACAACTC (22) | 111 |
| Mcp1 | GCTCAGCCAGATGCAGTTAA (20) | TCTTGAGCTTGGTGACAAAAACT(23) | 148 |
| Cxcl1 | TGTTGTGCGAAAAGAAGTGC (20) | ACACGTGCGTGTTGACCATA (20) | 160 |
| Ywhaz | TTACTTGGCCGAGGTTGCT (19) | TGCTGTGACTGGTCCACAAT (20) | 60 |

Supplementary Table 1. The primer sequences.
