# Supplementary material for: Cellular senescence contributes to radiation-induced hyposalivation by affecting the stem/progenitor cell niche
Source: Cell Death Dis. 2020 Oct 14;11(10):854. doi: 10.1038/s41419-020-03074-9 (PMC7566836; doi:10.1038/s41419-020-03074-9)
Supplement: Supplementary file 2 — Supplementary Table 1 legends [file 41419_2020_3074_MOESM2_ESM.docx]

Supplementary Table 1. The primer sequences.
