# Supplementary material for: Cellular senescence contributes to radiation-induced hyposalivation by affecting the stem/progenitor cell niche
Source: Cell Death Dis. 2020 Oct 14;11(10):854. doi: 10.1038/s41419-020-03074-9 (PMC7566836; doi:10.1038/s41419-020-03074-9)
Supplement: Supplementary file 3 — Supplementary figure legends [file 41419_2020_3074_MOESM3_ESM.docx]

**Supplementary figure legends**

**Supplementary Fig. 1 P16 and BCL-2 expression in salivary glands. a)** Representative images of p16 (brown) of human controls (63 and 85 years old) and radiation damaged 45-year-old salivary glands. Scale bar, 50 µm.**b)** Representative image of BCL-2 expression in control human salivary gland main ducts. Scale bar, 50 µm. Note the staining near the nuclear envelope of cells in the basal layer, suggested to be the salivary gland stem cells^21^.

**Supplementary Fig. 2 Cell cycle distribution and γH2AX expression** **of salivary gland organoid derived cells.** DNA content-based cell cycle histogram of cells derived from **a**) D5 control, **b**) D12 control, and **c**) IR (D7 after irradiation with 7 Gy) organoids. N=6 / group. **d)** Experimental design for panel . **e)** γH2AX expression in nuclei (DAPI) of irradiated organoids at different time points after irradiation, indicating DNA damage response activation. Scale bar, 25 µm.

**Supplementary Fig. 3 Salivary gland organoids CD24/CD29** **stem cell enrichment factor expression after irradiation. a)** Experimental design. **b)** Representative images and flow cytometric gating strategy used to separate CD24- and CD29- stained organoid-derived cells into four subsets: CD24^lo^/CD29^lo^; CD24^hi^/CD29^lo^, CD24^hi^/CD29^med^, CD24^hi^/CD29^hi^. **c-f)** Percentage of organoid derived cells from the different CD24/CD29 subsets. N=3 / group. Data are mean ± s.e.m. *P<0.05, **P<0.01, ***P<0.001. Student’s t-test.

**Supplementary Fig. 4 Irradiation response of salivary gland organoids.** **a**) SA-β-gal staining was performed on whole mount organoids collected at the indicated times. Representative images of D5 control, D12 control and IR organoids. Scale bar, 50 μm. **b**) Experimental design for panel c. **c**) Cell number analysis, single cells derived organoids from p16-3MR mice were (sham-) irradiated at D5. At D12 cell numbers were quantified. N=3 / group. **P<0.01, Wilcoxon signed-rank test.

**Supplementary Fig. 5 Effect of GCV on salivary gland organoids.** **a**) Experimental design for panels b-c. Single cells were seeded in matrigel at day 0 (D0) and cultured for 7 days to form secondary organoids (D7). Vehicle or GCV (10 μg/ml, 20 μg/ml, 50 μg/ml, 100 μg/ml and 200 μg/ml) were added to the medium immediately after cell seeding. GCV was refreshed every other day for 6 days. Organoid formation efficiency (OFE) percentage was quantified at D7. **b**) Representative images of D7 organoids in culture. **c**) Quantification of OFE at D7. N=3 / group. Throughout, data are means ± s.e.m. *P<0.05, **P<0.01, ***P<0.001, ****P<0.0001. Student’s t-test. Scale bar, 200 μm.

**Supplementary Fig. 6 Effects of ABT263 on proliferating and senescent salivary gland stem cells.** **a**) Experimental design for panels b-c. Single cells were seeded in matrigel (D0) and cultured for 7 days to form organoids (D7). Vehicle or 0.313 μM ABT263 were administrated immediately after cell seeding for 1, 5, 10 and 24 h, where after the media was refreshed. OFE percentage was quantified at D7. **b**) Quantification of OFE at D7. **c**) Representative images of D7 organoids in culture treated with vehicle or 0.313 μM ABT263 for 1, 5, 10 and 24 h. **d**) Experimental design for panels e-f. Organoids were treated with 0.313 μM ABT263 for 1 h at day 5 (D5). Single cells were passaged to check OFE at day 12 (D12). **e**) Quantification of OFE at D12. **f**) Representative images of D12 organoids in culture treated with vehicle or ABT263 for 1 h. **g**) Experimental design for panels h-i. Single cells were seeded in matrigel (D0) and cultured to generate organoids. Organoids were (sham-)IR-treated at day 5 (D5), and subsequently (sham-) treated with 0.313 μM ABT263 for 1, 5, 10 and 24 h at day 12. Single cells were passaged to check OFE at day 19 (D19) (**h**). **i**) Representative images of organoids at D19. N=3 / group, **P<0.01, ***P<0.001. Student’s t-test (b, e), Wilcoxon signed-rank test (h), Scale bar, 200 μm.
