# Supplementary figures and images for: Cellular senescence contributes to radiation-induced hyposalivation by affecting the stem/progenitor cell niche
Source: Cell Death Dis. 2020 Oct 14;11(10):854. doi: 10.1038/s41419-020-03074-9 (PMC7566836; doi:10.1038/s41419-020-03074-9)

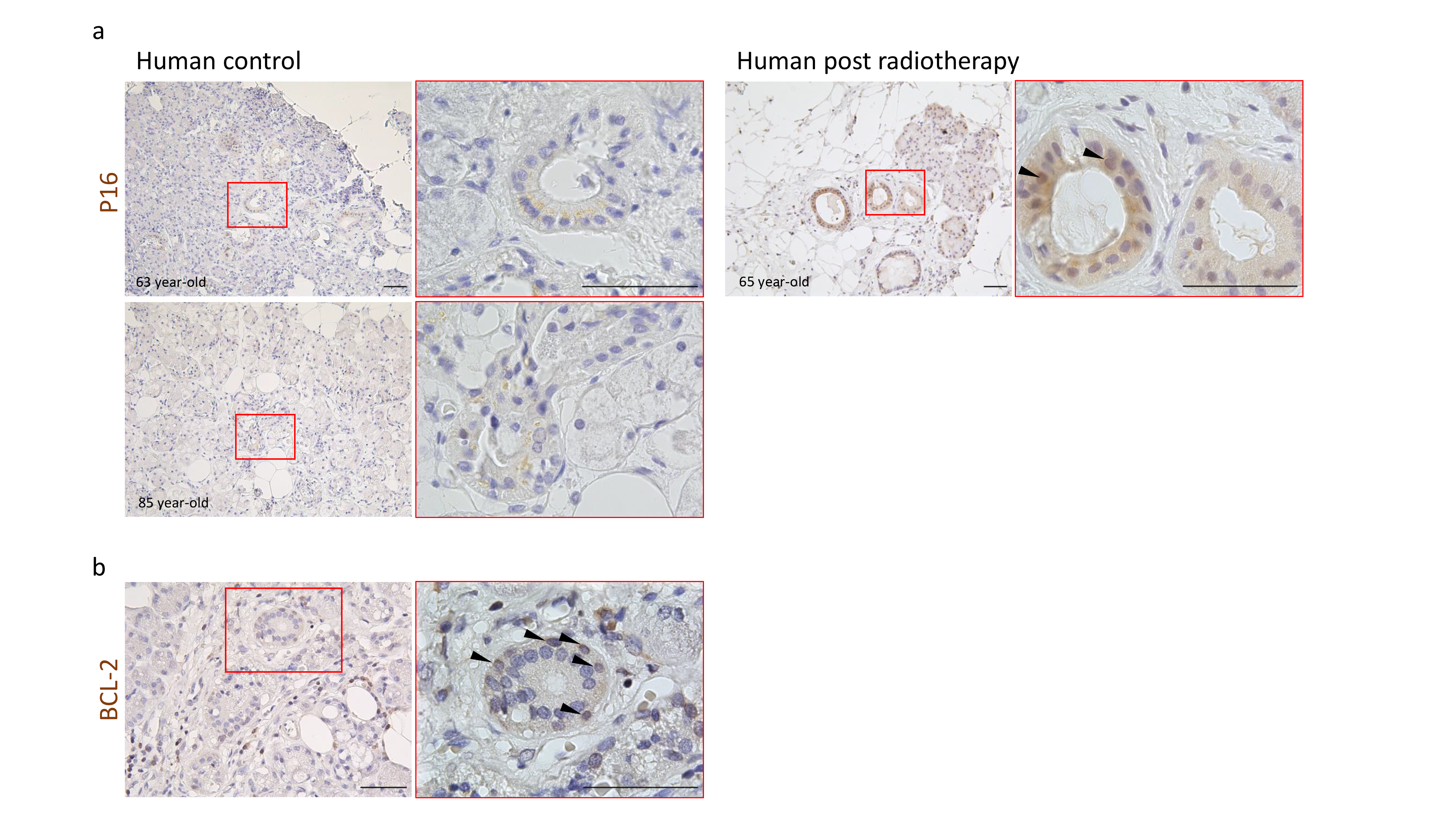

Supplement: Supplementary file 4 — Supplementary figure 1 [file 41419_2020_3074_MOESM4_ESM.tif]

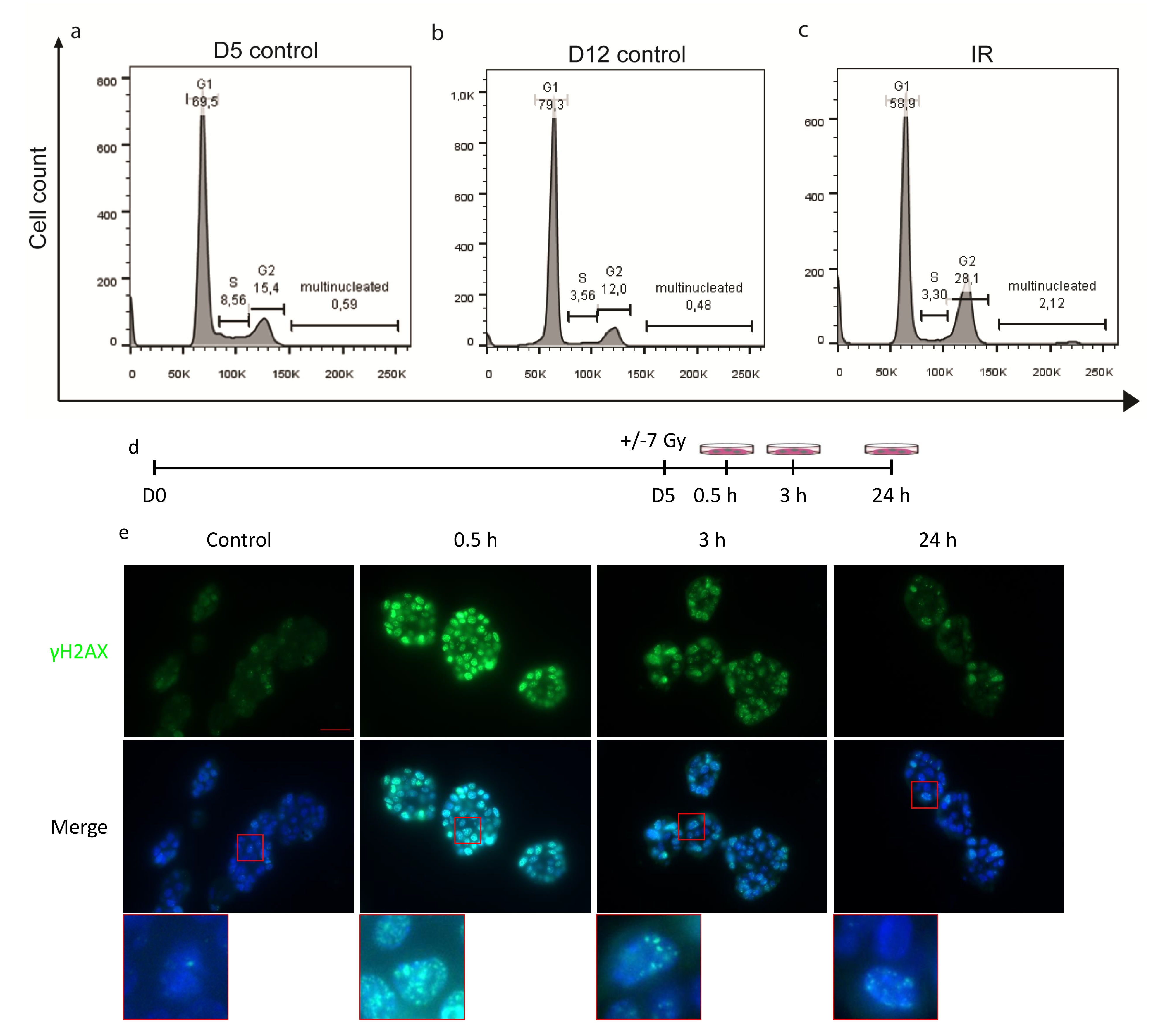

Supplement: Supplementary file 5 — Supplementary figure 2 [file 41419_2020_3074_MOESM5_ESM.tif]

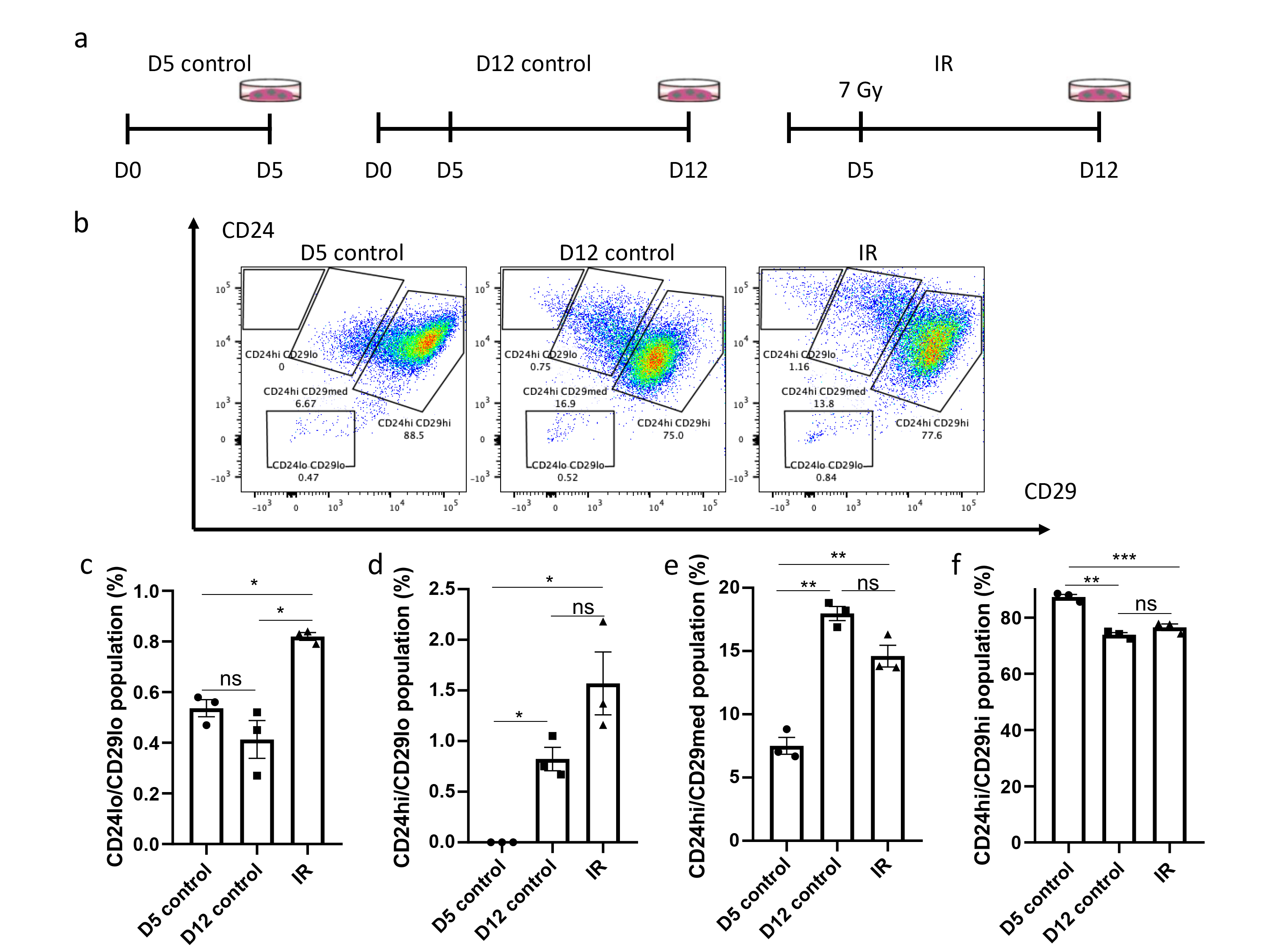

Supplement: Supplementary file 6 — Supplementary figure 3 [file 41419_2020_3074_MOESM6_ESM.tif]

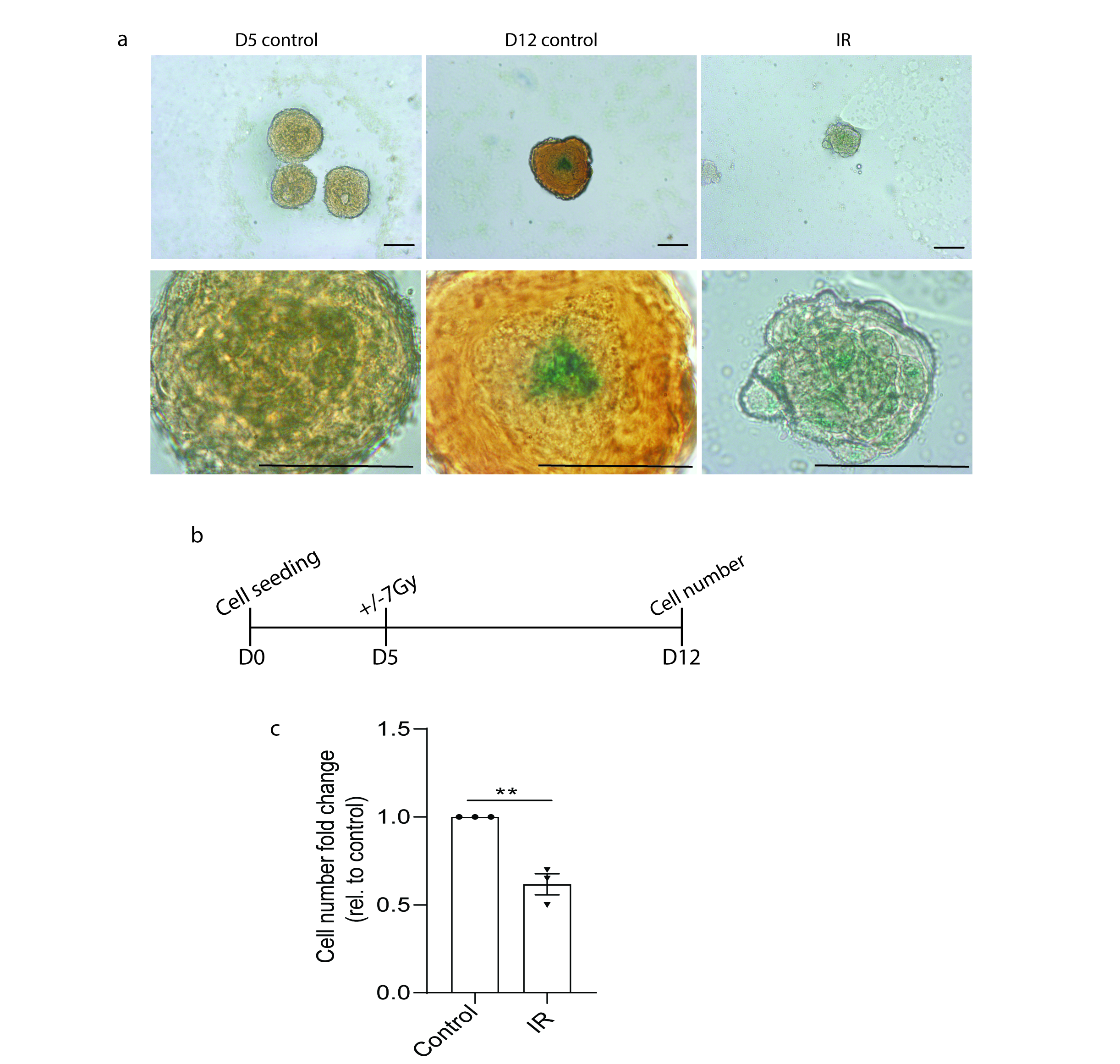

Supplement: Supplementary file 7 — Supplementary figure 4 [file 41419_2020_3074_MOESM7_ESM.tif]

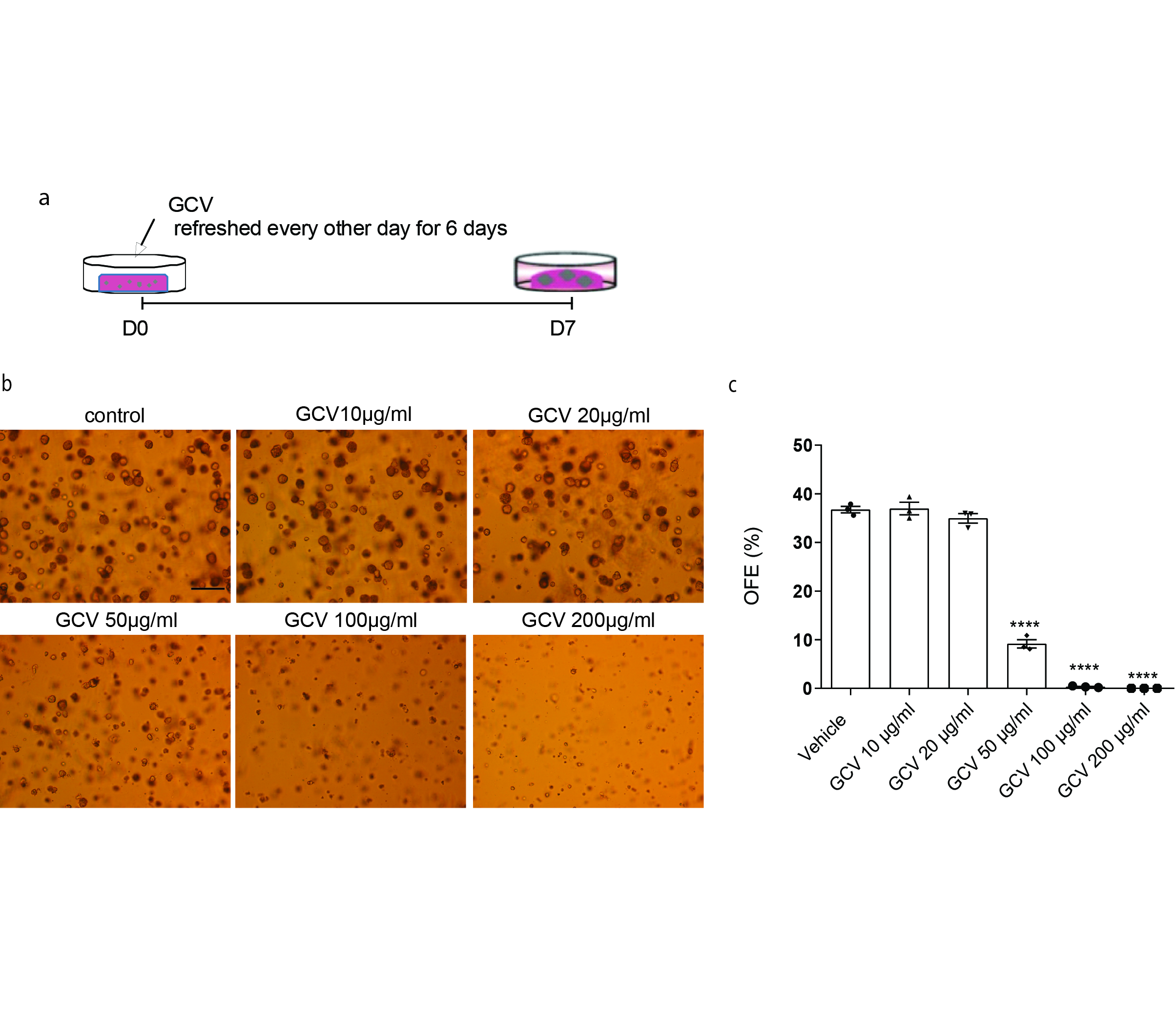

Supplement: Supplementary file 8 — Supplementary figure 5 [file 41419_2020_3074_MOESM8_ESM.tif]

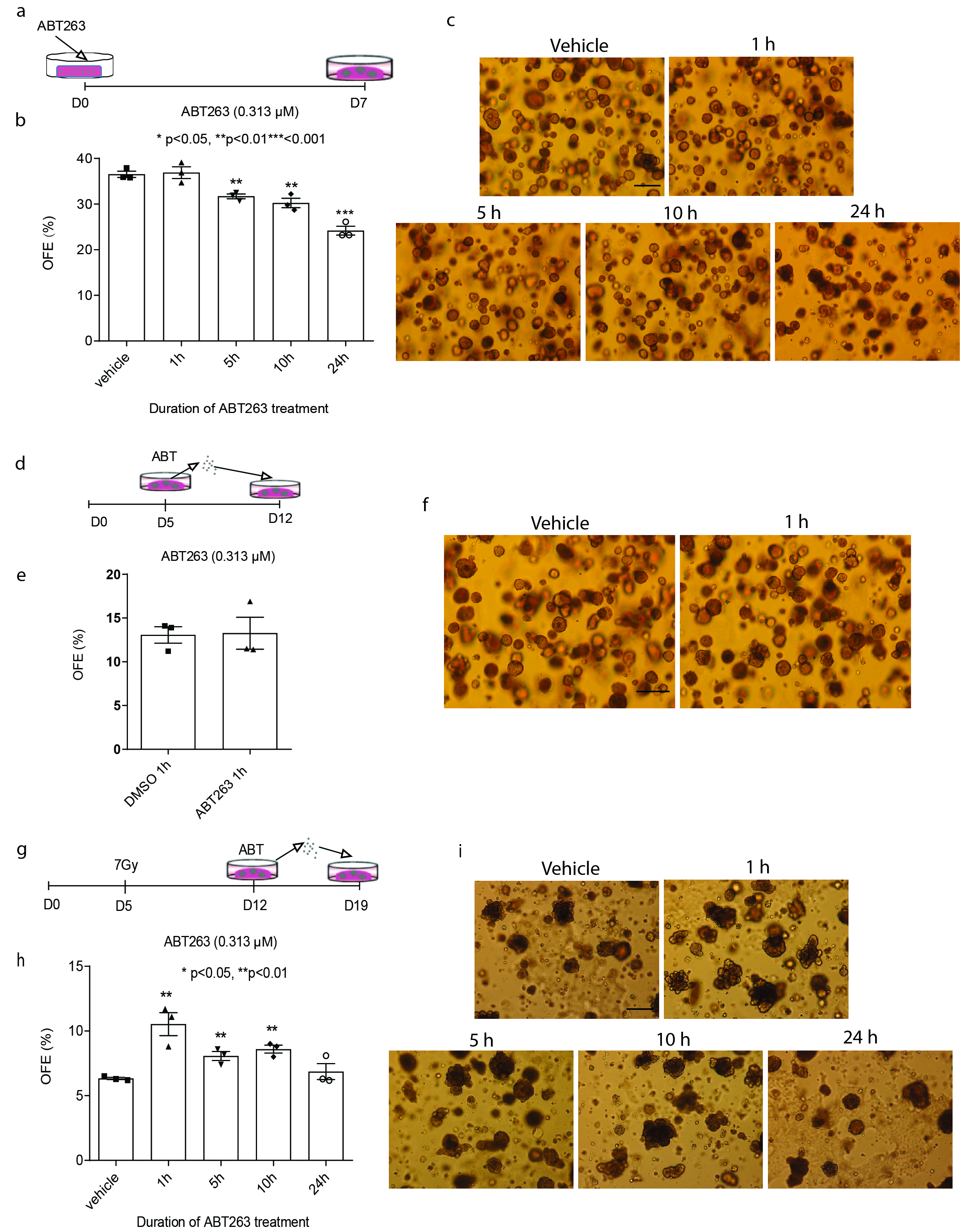

Supplement: Supplementary file 9 — Supplementary figure 6 [file 41419_2020_3074_MOESM9_ESM.tif]
